# Searching for treatments for non-G12C-*KRAS* mutant cancers

**DOI:** 10.1038/s41416-021-01357-2

**Published:** 2021-04-15

**Authors:** Christina Guo, Udai Banerji

**Affiliations:** 1 The Institute of Cancer Research, London, UK; 2 The Royal Marsden Hospital NHS Foundation Trust, London, UK

**Keywords:** Drug development, Medical research

## Abstract

*KRAS* mutations drive a wide variety of cancers. Drugs targeting the protein product of *KRAS*^G12C^ mutations are currently being evaluated show preliminary efficacy in clinical trials. A clinical trial of VS-6766, a dual RAF–MEK inhibitor, has reported early single agent activity in non-G12C mutated *KRAS* driven cancers.

## Main

*KRAS* mutations are commonly associated with a range of solid tumours and haematological malignancies.^1^ Initial attempts to drug KRAS proved challenging due to the high affinity of KRAS to GTP and the relative abundance of GDP/GTP in human cellular tissue.^2^ This led a plethora of efforts to target downstream effectors such as RAF, MEK, PI3K, AKT, mTOR and the combination of these signalling nodes,^1–3^ but these approaches have not led to registration of a drug or drug combinations in the setting of *KRAS* mutant (*KRAS*^M^) cancers. There has however been significant progress in developing drugs directly targeting protein products of specific subsets of *KRAS* mutations i.e. *KRAS*^G12C^. These inhibitors (sotorasib and adagrasib) have shown promising activity in non-small cell lung cancer (NSCLC) with *KRAS*^G12C^ mutations^4,5^ and will no doubt be further explored in registration enabling studies.

Guo et al. reported a Phase 1b dose-escalation, basket expansion study of a dual RAF–MEK inhibitor, VS-6766 (previously known as CH5126766 and RO5126766), which showed promising anti-tumour activity in patients with solid tumours and multiple myeloma harbouring non-G12C-*KRAS* mutations.^6^ There are several interesting aspects to this study.

Firstly, the drug has interesting pharmacological properties. It is a MEK inhibitor but also blocks in-complex (CRAF–MEK) phosphorylation of MEK,^7^ leading to reduction of phosphorylation of both MEK and ERK as demonstrated in pre- and post-treatment biopsies. This is distinct to a reduction of phosphorylation of ERK but not MEK caused by currently licensed MEK inhibitors. The inhibition of signalling in two distinct nodes in the MEK–ERK signalling network by a single drug may explain the preliminary single agent efficacy of VS-6766 reported.

Secondly, the authors have exploited an unusually long half-life of the drug of approximately 50 hours (which is significantly longer than other RAF or MEK inhibitors) to run pharmacokinetic simulations to predict drug concentrations and design a twice a week dosing schedule. The highly intermittent twice a week schedule established in the trial enables patients to tread the fine line between efficacy and toxicity. Further, a dose modification strategy to drop to three-weeks on-one week off rather than reducing the dose of the drug is different from dose modification strategies commonly used in combination studies of targeted agents.^8^ This reflects previous preclinical studies by the group where they had studied the importance of maximal inhibition of MEK signalling in *KRAS*^M^ models.^9^ The intermittent schedule has significantly improved the therapeutic index of the drug compared with previous studies that explored continuous dosing schedules.^10^

Finally, the authors have demonstrated single agent activity in a variety of *KRAS*^M^ cancers in a cohort of heavily pre-treated patients. Of note, they have demonstrated partial responses in 3/10 patients with *KRAS*^M^ NSCLC as a single agent and interestingly two of the three patient who responded had *KRAS*^G12V^ mutations. Given there are no KRAS targeted therapies for *KRAS*^G12V^ mutations, VS-6766 could be explored further in this disease space both as a single agent or in combination. Of note, 3/5 patients with *RAS/RAF* mutations with gynaecological cancers responded to treatment. There is considerable excitement and activity of MEK inhibitors (trametinib and binimetinib) in low grade serous ovarian cancer (LGSOC).^11,12^ The combination study of VS-6766 with defactinib have shown promising activity in Phase 1 studies with expansions in LGSOC^13^ and randomised a Phase 2 trial is exploring the activity of the combination in LGSOC is ongoing (NCT04625270). Further, an interesting response was seen in patient with *KRAS*^G12V^ driven multiple myeloma. While anecdotal responses of MEK inhibitors have been noted in myeloma,^14^ this further confirms response of this agent in an independent cancer type driven by *KRAS* mutations.

**Fig. 1:**
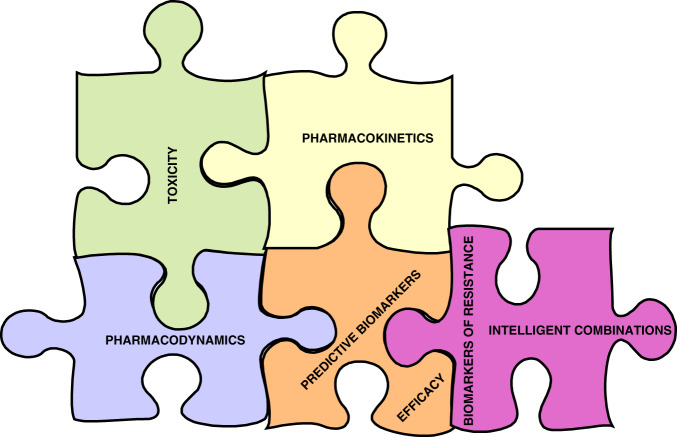
The Pharmacological Audit Trial. Crucial elements in the pharmacological audit trail that are key to optimise dosing schedules and use predictive biomarkers to accelerate early clinical development.

Attention to detail of pharmacokinetics, pharmacodynamics, toxicity and predictive biomarkers of efficacy are part of the pharmacological audit trail and remain critical to successful development of targeted therapy^15^ (Fig. 1). The trial by Guo et al.^6^ has defined a backbone of a highly intermittent schedule of novel RAF–MEK inhibitor VS-6766. Multiple early Phase clinical trials of combinations with this VS-6766 are currently ongoing with agents such as everolimus (NCT02407509) and defactinib (NCT03875820). Randomised studies of the combination of VS-6766 and defactinib to explore efficacy of the combination in *KRAS*^M^ LGSOC (NCT04625270) and NSCLC (NCT04620330) are currently ongoing.

## Data Availability

Not applicable.
